# Integrated Analysis of Metatranscriptome and Amplicon Sequencing to Reveal Distinctive Rhizospheric Microorganisms of Salt-Tolerant Rice

**DOI:** 10.3390/plants14010036

**Published:** 2024-12-26

**Authors:** Wenna Meng, Zhenling Zhou, Mingpu Tan, Anqi Liu, Shuai Liu, Jiaxue Wang, Zhiguang Sun, Yiluo Tan, Yan Liu, Baoxiang Wang, Yanming Deng

**Affiliations:** 1 College of Life Sciences, Nanjing Agricultural University, Nanjing 210095, China; 2022116038@stu.njau.edu.cn (W.M.); tempo@njau.edu.cn (M.T.); anqiliu@stu.njau.edu.cn (A.L.); 2021816135@stu.njau.edu.cn (S.L.); 2023816123@stu.njau.edu.cn (J.W.); 2 Lianyungang Academy of Agricultural Sciences, Lianyungang 222000, China; zhouzl13716@163.com (Z.Z.); zhiguangsun@126.com (Z.S.); 15805132001@163.com (Y.T.); ly516.bester@163.com (Y.L.); 3 Institute of Leisure Agriculture, Jiangsu Academy of Agricultural Sciences, Nanjing 210014, China

**Keywords:** metatranscriptome, plant–microbe interaction, salt tolerance, rice

## Abstract

Salt stress poses a significant constraint on rice production, so further exploration is imperative to elucidate the intricate molecular mechanisms governing salt tolerance in rice. By manipulating the rhizosphere microbial communities or targeting specific microbial functions, it is possible to enhance salt tolerance in crops, improving crop yields and food security in saline environments. In this study, we conducted rice rhizospheric microbial amplicon sequencing and metatranscriptome analysis, revealing substantial microbiomic differences between the salt-tolerant rice cultivar TLJIAN and the salt-sensitive HUAJING. Fungal taxa including *Hormiactis*, *Emericellopsis*, *Ceriosporopsis*, *Dirkmeia*, and *Moesziomyces* predominated in the rhizosphere of salt-tolerant rice, while bacterial genera such as *Desulfoprunum* and *Hydrogenophaga* exhibited notable differences. Metatranscriptomic analysis identified 7192 differentially expressed genes (DEGs) in the two rice varieties, with 3934 genes being upregulated and 3258 genes being downregulated. Enrichment analyses in KEGG and GO pathways highlighted the majority of DEGs were associated with the “two-component system”, “sulfur metabolism”, and “microbial metabolism in diverse environments”. The interaction network of DEGs and microbial taxa revealed upregulation of transporters, transcriptional factors, and chaperones, such as ABC transporters and chaperonin GroEL, in the rhizosphere microbiomes of salt-tolerant varieties. Our multi-omics network analysis unveiled that fungi like *Ceriosporopsis* and *Dirkmeria*, along with bacteria such as *Desulfoprunum*, *Rippkaea*, and *Bellilinea*, showed a positive correlation with flavonoid synthesis in salt-tolerant rice. This study provides an in-depth exploration of the distinctive microbial communities associated with the rhizosphere of salt-tolerant rice varieties, shedding light on the complex interactions between these microbial consortia and their host plants under stress conditions.

## 1. Introduction

One of the most pressing global soil-related challenges is salinization, a phenomenon that significantly hampers crop yield and quality [1]. With over 800 million hectares of arable land worldwide already impacted by soil salinity, the prevalence of alkali soil is projected to escalate in the foreseeable future. Salt stress has the potential to slash crop yields by 50% or more, triggering adverse ecological and socio-economic repercussions [2]. Breeders have made numerous attempts in salt tolerant plant breeding, the adaptability of plants is not solely determined by intrinsic factors but also by the intricate interplay with diverse environmental biological components [3,4]. Soil microorganisms play a pivotal role in nutrient cycling, soil fertility maintenance, and enhancing crop productivity, as highlighted in multiple research studies [5,6]. Rice is one of the most salt-sensitive cereal crops, especially in the early stages of seedlings. Breeding salt-tolerant rice varieties will greatly contribute to the global food security [7]. In recent years, a combination of traditional breeding methodologies and the integration of beneficial microorganisms has emerged as a promising approach to enhance crop salt tolerance [8].

Plants have evolved intricate mechanisms to thrive in saline–alkali environments by engaging in continuous interactions that extend to influence soil microorganisms through dynamic host-microbe relationships [9]. The rhizosphere, acting as a specialized microorganism habitat, serves as a hotspot for microbial activity crucial for plant health [10]. Microbiomes play a crucial role in supporting plant growth, enhancing stress tolerance, and broadening the plant’s metabolic capabilities by introducing new defense mechanisms [11]. The rhizospheric microbiome acts as a salinity-alleviating agent, which benefits plants directly by controlling nutrient attainment and phytohormone equilibrium or indirectly by inducing signaling pathways in the host to mitigate salt stress [12,13,14,15]. Therefore, this intricate web of interactions underscores the significance of the plant microbiome, often likened to the “second genome” of plants, in bolstering plant vigor, development, and immunity [16]. 

The soil microbiome has demonstrated significant potential in enhancing plant resilience against abiotic stress, indicating its promising role as a sustainable and effective approach [2]. Specifically, the diverse array of microbial communities intricately intertwined with plant roots plays a pivotal role in enabling plants to adapt to challenging saline–alkali environments [17]. Plant growth-promoting rhizobacteria (PGPB) have been recognized as vital biological tools for mitigating salt stress in plants through multiple mechanisms [15,18,19]. PGPB colonize plants and enhance salt tolerance in host plants by prompting the accumulation of antioxidants and osmoprotectants, ion homeostasis, induced systemic tolerance, and regulation of the stress response genes [14,15].

Many PGPB species are equipped with secretory systems and can produce antibacterial compounds, including antibiotics, volatile organic compounds, and lytic enzymes, empowering them to impede the proliferation of potential plant pathogens [20]. In addition, various fungi in plant roots, such as mycorrhizal fungi and endophytic fungi, can alleviate toxic symptoms in plants under abiotic stress by promoting root growth, enhancing nutrient absorption, activating antioxidant enzyme activity, and regulating hormone levels [21,22]. Salt stress exerts a significant impact on the diversity of rhizosphere bacteria and the composition of rhizosphere metabolites, with specific bacterial groups potentially playing pivotal roles in mitigating salt stress in seawater rice SR86 [23]. Recent studies have shown that the specific enrichment of breed-driven rhizosphere microbiomes may be a key factor in determining their salt tolerance [24]. Therefore, the composition and structure of rhizosphere microbial communities are influenced by soil type, while the host genotype contributes to the specific species composition and abundance [25]. In recent years, significant advancements in microbial metagenome and metatranscriptome sequencing have revolutionized the exploration of microbial community structure diversity offering valuable insights into the intricate mechanisms underlying plant stress resistance [26,27]. By leveraging these high-throughput sequencing analyses, researchers can effectively showcase the pivotal role of rhizosphere microorganisms in enhancing plant salt tolerance [28]. However, the amplicon sequencing and metatranscriptome analysis of rhizospheric microbiota associated with rice varieties differed in salt tolerance and is still in its infancy. 

This study delves into the rhizosphere microbiomic differences between salt-tolerant and salt-sensitive rice varieties. The integrative microbial amplicon sequencing and metatranscriptome analysis revealed the upregulation of transporters, transcriptional factors, and chaperones in the rhizosphere fungi and bacteria of salt-tolerant rice. Further multi-omics analysis of associations among rice genes, metabolites, and microorganisms revealed that Fungi such as *Ceriosporopsis* and *Dirkmeria*, and bacteria such as *Desulfoprunum*, *Rippkaea*, and *Bellilinea* were positively correlated with the synthesis of flavonoids in salt-tolerant rice. This comprehensive analysis has revealed key insights into how salt-tolerant rice varieties foster a unique microbial environment, which benefits plants directly or indirectly by exerting a positive effect on rice salt tolerance.

## 2. Materials and Methods

### 2.1. Sample Collection and Processing

Rice rhizosphere soil samples were collected from four varieties of japonica rice (*Oryza sativa* ssp. japonica), including two salt-tolerant varieties (Lianjian 5 (TLJIAN) and Yandao 16Z38 (TYDAO)) and two salt-sensitive varieties (Huajing 5 (HJING) and Lianjing (LJING)). The rice plants were cultivated in the Qingkou Salt Field Experimental Base (salt content in soil > 0.5%) of Jiangsu Lianyungang Academy of Agricultural Sciences in 2022. Seedlings were transplanted to the field on 15 June, and irrigated with fresh water (salt content ~0.15%) to maintain a relatively stable soil salt content throughout the entire growth period, as described previously [29]. On 28 August, at the rice booting stage, rice rhizosphere soil samples were collected and placed in sterilized 5 ml centrifuge tubes, and stored in a −80 °C refrigerator before extracting microbial DNA and RNA, with 3 replicates set for each sample. The amplicon sequencing materials were the soil samples from the rhizosphere of the four rice varieties, and the metatranscriptome sequencing materials were from the rhizosphere of HJING and salt-tolerant rice TLJIAN.

### 2.2. DNA Extraction and PCR Amplification

The DNA was extracted from rice rhizosphere soil samples by using the E.Z.N.A.^®^ Soil DNA Kit (Omega BioTek, Norcross, GA, USA), according to the manufacturer’s protocol. After extraction, the V4-V5 region of the bacterial 16S ribosomal RNA gene was amplified using the primers 799F: 5′-AACMGGATTAGATACCCKG-3’ and 1193R: 5’-ACGTCATCCCCACCTTCC-3′, along with the non-coding ITS regions of fungal rRNA genes using the primers ITS1F: 5′-CTTGGTCATTTAGAGGAA GTAA-3′ and ITS2R: 5′-GCTGCGTTCTTCATCGATGC-3′. The PCR reaction mixture was incubated at 95 °C for 2 min, followed by 27 cycles at 95 °C for 30 s, 55 °C for 30 s, and 72 °C for 60 s, and a final extension at 72 °C for 5 min. PCR reactions were performed in a triplicate 20 μL mixture containing 4 μL of 5 × FastPfu Buffer, 2 μL of 2.5 mM dNTPs, 0.8 μL of each primer (5 μM), 0.4 μL of FastPfu Polymerase, and 10 ng of template DNA. The pooled PCR products were cleaned using the AxyPrep DNA Gel Extraction Kit (Axygen Biosciences, Union City, CA, USA) according to the manufacturer’s instructions.

### 2.3. Amplicon (16S rRNA and ITS) Sequencing and Annotation

The purified PCR products were quantified using Qubit 3.0 (Life Invitrogen) and every 24 amplicons with different barcodes were mixed equally [30]. The pooled DNA products were used to construct the Illumina Pair-End library, and then the amplified library was sequenced on the Illumina MiSeq platform (Shanghai BIOZERON Co., Ltd., Shanghai, China) according to the standard protocols. 

The sequencing data were analyzed with bioinformatics tools as follows. Initially, primer sequences were removed from the sample FASTQ files [31]. The remaining sequences were clustered into operational taxonomic units (OTUs) with a 98.65% similarity cutoff using UPARSE (version 7.1.6) and chimeric sequences were identified and removed using UCHIME [32,33]. The Ribosomal Database Project (RDP) classifier uses a 70% confidence threshold to analyze the phylogenetic relationships of each 16S rRNA gene sequence against the silva (SSU132) 16SrRNA database. 

Based on the Kyoto Encyclopedia of Genes and Genomes (KEGG) database, the functional alteration of microbiota in different samples was predicted by the Phylogenetic Investigation of Communities by Reconstruction of Unobserved States (PICRUSt) (http://picrust.github.io/picrust/tutorials/genome_prediction.html (accessed on 16 September 2024)) program. The obtained OTU was used to generate BIOM files, which were formatted as input to PICRUSt. OTU abundances were mapped to Greengenes OTU IDs as input to infer the functional changes in the microbiota. 

### 2.4. Metatranscriptome Sequencing and Data Processing

Total RNA was extracted from rhizosphere soil samples using the TRIzol^®^ Reagent. DNase treatment was conducted using DNase I (Takara). Ribosomal RNA was depleted in these samples using the Ribo Zero TM rRNA Removal Kit. Metatranscriptome libraries were prepared using the TruSeq TM Stranded Total RNA Sample Preparation Kit from Illumina (San Diego, CA, USA). cDNA synthesis, end repair, A-base addition, and ligation of the Illumina-indexed adaptors were performed according to the official protocol. Then metatranscriptome libraries were sequenced using the Illumina HiSeq 2500 platform in Shanghai Biozeron Technology Co., Ltd. (Shanghai, China). Read trimming and quality control were performed using Trimmomatic (http://www.usadellab.org/cms/?page=trimmomatic (accessed on 28 September 2024)) [34].

The clean reads were aligned to the SILVA SSU (16S/18S) and SILVA LSU (23S/28S) databases to remove rRNA-related reads using SortMeRNA (https://github.com/sortmerna/sortmerna (accessed on 28 September 2024)). Non-redundant UniGene catalogs were constructed with 95% identity and 90% coverage by CD-HIT (https://www.bioinformatics.org/cd-hit (accessed on 28 September 2024)) [35]. All UniGene catalogs were searched against NCBI non-redundant proteins (NR), String, and Kyoto Encyclopedia of Genes and Genomes (KEGG) databases using BLASTp, and their functions were inferred by the annotation of the sequence with the highest similarity. GO annotations were performed by blast2go (https://www.blast2go.com (accessed on 6 October 2024)) [36]. Metabolic pathway analysis was performed using the KEGG database (https://www.kegg.jp/ (accessed on 6 October 2024)) [37].

To identify differentially expressed genes (DEGs) between different samples, Salmon (https://github.com/COMBINE-lab/salmon (accessed on 16 October 2024)) was used to calculate the expression levels of each transcript, and RSEM (http://deweylab.github.io/RSEM (accessed on 16 October 2024)) was used to quantify the abundance of genes and transcripts. In addition, functional-enrichment analysis was carried out using GO functional enrichment and KEGG pathway analysis via Goatools (https://github.com/tanghaibao/goatools (accessed on 16 October 2024)) and KOBAS (http://bioinfo.org/kobas (accessed on 16 October 2024)) to determine which DEGs were significantly enriched in metabolic pathways at Bonferroni-corrected *p*-value < 0.05 [38,39].

### 2.5. Integrative Multi-Omics Analysis and Statistics

All statistical analyses were performed in the R environment (v4.1.0, https://www.r-project.org (accessed on 28 October 2024)). Based on 16S and ITS sequences, UniFrac was used for beta diversity analysis and comparison with the principal component analysis (PCA) results [40]. Taxonomic classification of operational taxonomic units (OTUs) was performed to determine the composition and relative abundance of each taxon, with a 97% similarity cut-off threshold [41,42]. The linear discriminant analysis (LDA) effect size (LEfSe) was used to identify dominant microorganisms in the rhizosphere of rice with different salt tolerance [43]. A linear discriminant analysis (LDA) was conducted to evaluate the influence of biomarkers on significantly different groups based on LDA scores [44]. For the integrative multi-omics analysis, metabolomic data of rice with different salinity tolerances [29] were employed to calculate their correlation with rhizospheric microbiota. Pearson correlation coefficients (PCC) between DEGs and microorganisms, as well as metabolites were calculated to determine the correlation between them. A threshold of |PCC| > 0.8 was set to infer meaningful relationships. Important metabolites, critical genes, and dominant microorganisms were selected to construct the gene–metabolite–microorganism correlation network using Cytoscape v3.7.1 [45].

## 3. Results

### 3.1. Rhizospheric Microbiota Characteristics of Rice Cultivars with Different Salt Tolerance

**Figure 1:**
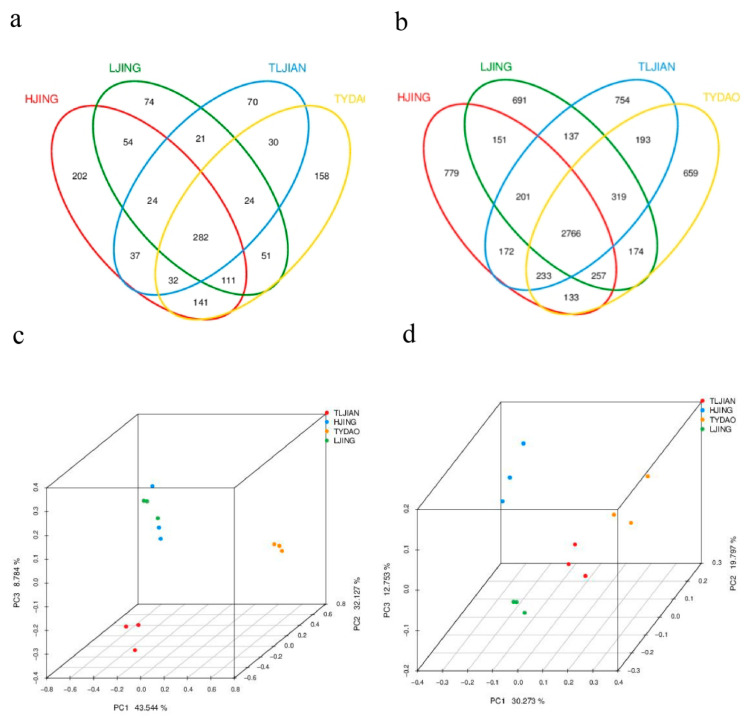
Comparative analysis of the microbiome composition based on amplifier sequencing 16S and ITS sequencing in the rhizospheres of four rice varieties with different salt tolerance. (**a**,**b**) Venn diagram of specific and shared fungal and bacterial OTUs. (**c**,**d**) Principal component analysis of rhizosphere microorganisms. The left figures (**a**,**c**) represent fungi, and the right figures (**b**,**d**) represent bacteria.

To explore the rhizosphere soil bacterial and fungal communities associated with salt tolerance, a comprehensive analysis was conducted through high-throughput sequencing of 16S rRNA and internal transcribed spacer (ITS) regions across 12 rhizosphere samples (from salt-tolerant rice varieties TLJIAN and TYDAO to salt-sensitive rice HJING and LJING). At the fungal level, a total of 30 OTUs were shared between the two salt-tolerant rice rhizospheric microbiota, and 54 OTUs were shared between the two salt-sensitive rice counterparts. Similarly, at the bacterial level, 193 OTUs were shared between the two salt-tolerant rice rhizospheric microbiota, while 151 OTUs were shared between the two salt-sensitive rice counterparts (Figure 1a,b).

To compare the rhizosphere microorganism structures between two distinct salt-tolerant rice varieties, the taxonomic abundance matrix was converted to the Bray–Curtis distance matrix and used for subsequent analysis. Principal coordinate analysis (PCoA) showed that in terms of fungal composition, the rhizosphere microbial compositions of HJING and LJING exhibited notable similarities. However, at the bacterial level, a marked divergence was observed in the composition of rhizosphere microorganisms among the four rice varieties (Figure 1c,d). 

### 3.2. Rhizospheric Microbial Biomarkers for Distinguishing Rice Salt Tolerance

To discern the indicator bacterial and fungal species in the rhizosphere soil of rice cultivars that differ in salt tolerance, we conducted a comprehensive taxonomic analysis of the microbial consortia associated with the rhizospheres of salt-tolerant rice TLJIAN and salt-sensitive HJING. Leveraging LEfSe analysis with stringent criteria (LDA score > 3 and *p* < 0.05 for fungi, LDA score > 2.5 and *p* < 0.05 for bacteria), we identified microbial biomarkers that distinctly characterized the rhizosphere microbiomes of rice varieties with different salt tolerance.

**Figure 2:**
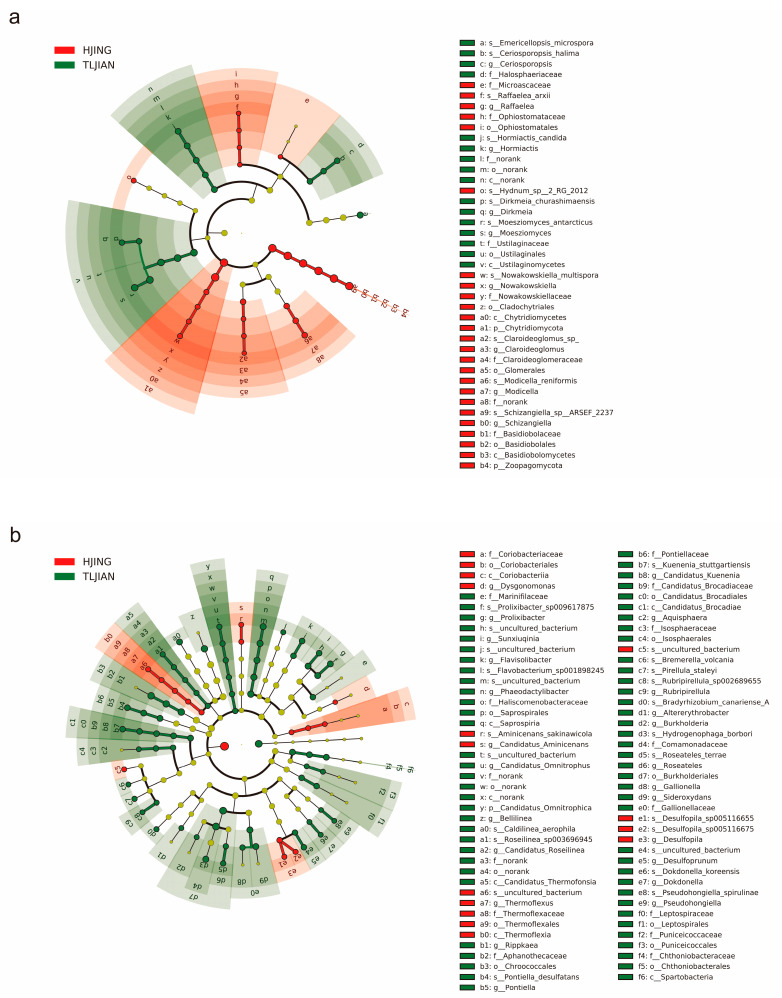
LEfSe analysis of fungi and bacteria differences in the rhizosphere of two rice varieties. (**a**) The different fungi taxa between salt-tolerant TLJIAN and salt-sensitive HJING rice rhizosphere. (**b**) The different bacteria taxa between salt-tolerant TLJIAN and salt-sensitive HJING rice rhizosphere. The circles from inner to outer layers represent the taxonomic levels from the phylum to species. The dots on the circles represent terms on the corresponding taxonomic level. The sizes of the dots indicate relative abundance. Coloring: yellow represents species with no significant difference, red for species enriched in the salt-sensitive HJING rhizosphere, and green for species enriched in salt-tolerant TLJIAN rhizosphere. The lowercase p, c, o, f, g, and s in front of the symbol “_” represent the phylum, class, order, family, genus, and species, respectively.

Consequently, 41 fungal taxa displayed a significant divergence in abundance between the two groups. At the genus level, *Hormiactis*, *Emericellopsis*, *Ceriosporopsis*, *Dirkmeia*, and *Moesziomyces* were enriched in the rhizosphere of salt-tolerant TLJIAN, while salt-sensitive HJING rhizosphere recruited six fungi genera, namely *Raffaelea*, *Hydnum*, *Nowakowskiella*, *Claroideoglomus*, *Modicella*, and *Schizangiella* (Figure 2a; Table 1). Moving to the bacteria realm, the LEfSe analysis revealed substantial abundance variations in 85 bacterial taxa of the two varieties that differed in salt tolerance (Figure 2b; Table S2). Notably, the bacterial composition in the rhizosphere of salt-tolerant TLJIAN was characterized by 12 predominant genera, prominently featuring *Desulfoprunum*, *Sideroxydans*, *Hydrogenophaga*, *Candidatus Kuenenia*, *Pontiella*, *Rippkaea*, *Caldilinea*, *Bellilinea*, *Phaeodactylibacter*, *Flavisolibacter*, *Sunxiuqinia*, and *Prolixibacter*, while the rhizosphere bacteria of salt-sensitive rice HJING were mainly concentrated in six genera, including *Desulfopila*, *Blastopirellula*, *Clostridium*, *Thermoflexus*, *Candidatus Aminicenans*, and *Dysgonomonas* (Figure 2b; Table 2) 

### 3.3. Comparative Analysis of the Rhizosphere Microbial Metatranscriptome

**Figure 3:**
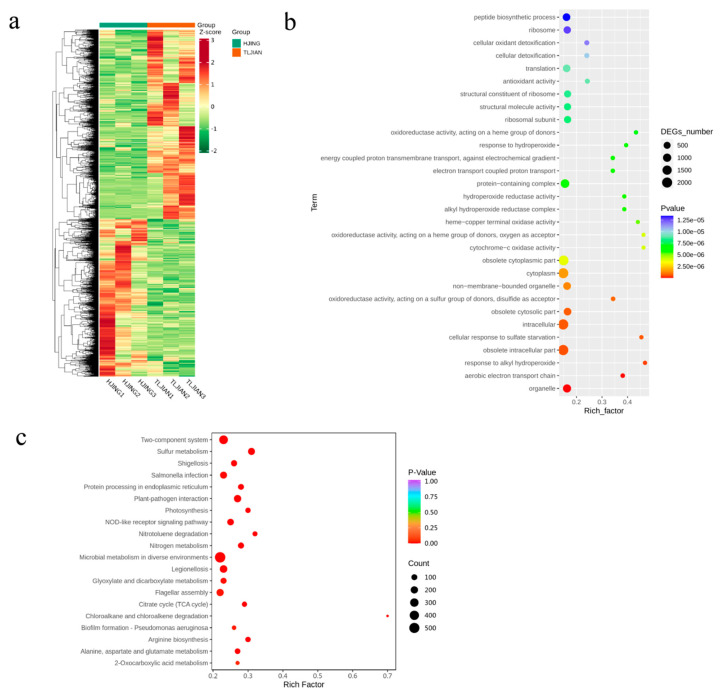
Microbial differentially expressed genes (DEGs) in the rhizospheric microbiome of salt-tolerant TLJIAN and salt-sensitive HJING rice. (**a**) Heat map of DEGs based on hierarchical clustering analysis. (**b**) Gene ontology (GO) enrichment analysis of DEGs. (**c**) Kyoto Encyclopedia of Genes and Genomes (KEGG) pathway analysis of DEGs.

Through metatranscriptomic analysis, 7192 DEGs were identified in the rhizospheric microbiome of salt-tolerant rice TLJIAN against salt-sensitive HJING, and 3934 genes were upregulated, whereas 3258 genes were downregulated in the rhizospheric microbiome of salt-tolerant rice (Figure 3a; Table S3). Moreover, 274 of 573 transporters and 113 of 165 transcriptional factors were upregulated in the rhizospheric microbiome of salt-tolerant rice (Tables S4 and S5). 

To gain insight into the biological pathway of potential transcripts, gene ontology (GO) and KEGG enrichment analysis of DEGs were performed. GO enrichment analysis indicated that DEGs were primarily enriched in “oxidoreductase activity”, “obsolete cytosolic part”, “response to alkyl hydroperoxide”, and “aerobic electron transport chain” (Figure 3b). Furthermore, the results of the KEGG pathway enrichment analysis suggested that DEGs were mainly enriched in “sulfur metabolism”, a “two-component system”, and “microbial metabolism in diverse environments” (Figure 3c). 

Regarding “Sulfur metabolism”, all Sox proteins (sulfur oxidation c-type cytochrome SoxA and SoxX, sulfur-oxidizing protein, and thiosulfate oxidation carrier protein SoxY and SoxZ) were uniformly upregulated in the rhizospheric microbiome of salt-tolerant rice. For the “two-component system” category, all chemotaxis proteins, phosphate ABC transporter substrate-binding proteins PstS and PhoT, sugar ABC transporters, and two-component system response regulators OmpR and QseB were uniformly upregulated in the rhizospheric microbiome of salt-tolerant rice. This was also the case for “Microbial metabolism in diverse environments” where nitrate reductase [*Hydrogenophaga flava*] and nitrogenase were uniformly upregulated. Moreover, over 30 DEGs associated with nutrient acquisition, hormone synthesis, osmolyte production, and antioxidant defense were identified in the reported genomes of saline soil PGPB [14]. Particularly, DEGs in the ‘Nitrogen fixation’ category such as bifunctional nitrogenase iron-molybdenum cofactor biosynthesis protein NifEN, nitrogenase molybdenum-iron protein, and nitrogen fixation protein FixH, were uniformly upregulated in rhizospheric microbiome of salt-tolerant rice, and this was also case for arginine decarboxylase and trehalose-phosphatase in the ‘osmolyte production’ category. Furthermore, the upregulated indole pyruvate ferredoxin oxidoreductase in the ‘IAA production’ category and superoxide dismutase and peroxiredoxin in the ’antioxidant defense’ category were also identified in bacteria enriched in salt-tolerant rice rhizosphere (Table 3 and Table S3).

These enrichment results indicated that the salt-responsive rhizospheric microbial DEGs have a potential role in salt stress regulation in rice.

### 3.4. Joint Analysis of the Rhizosphere Microbial Community Structure and Metatranscriptome

**Figure 4:**
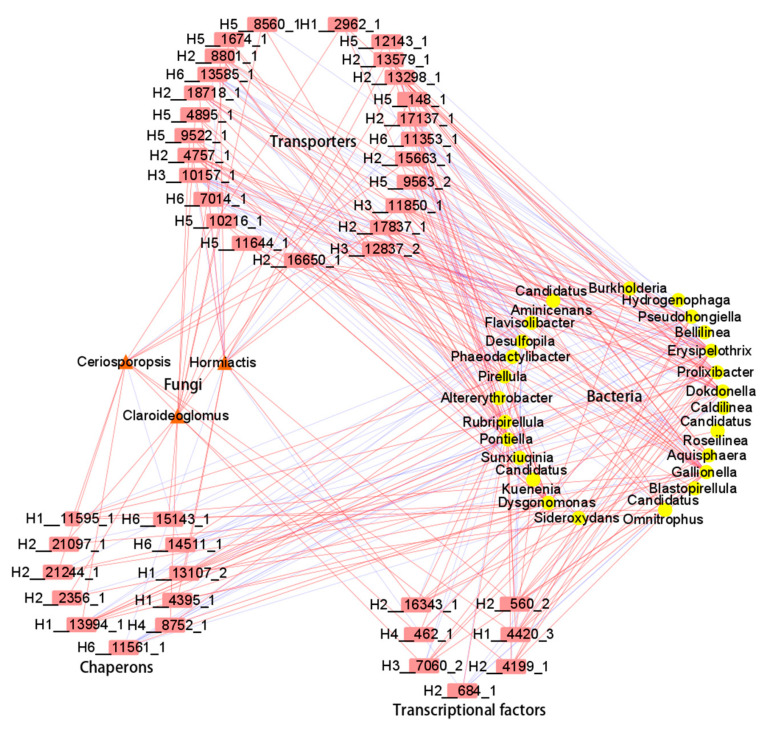
A metatranscriptome and microbiome network derived from comprehensive gene–microorganism associations. Rectangles represent genes, circles represent bacteria, and triangles represent fungi. The red and blue lines represent positive and negative correlations, respectively; the thickness of the line indicates the strength of the correlation. The thicker the line, the stronger the correlation. The detailed correlation data of genes and microorganisms are presented in Table S6.

Through a conjoint integration of metatranscriptome and microbiome data, the complex regulatory network for DEGs and microbial changes was revealed to understand the different mechanisms of salt stress response between the two types of rice. By examining gene–microbial associations with significant coefficient correlation (|PCC| > 0.90 and *p*-value < 0.05, Table S6), a total of 92 DEGs were highly correlated with salt-tolerant rice rhizosphere enriched microbiota, including 25 bacteria and three fungi (Claroideoglomus, Ceriosporopsis and Hormiactis). Specifically, 25 genes were annotated as transporters, 15 genes as chaperons, and seven genes as transcriptional factors (Table 4). Moreover, the transporters, transcriptional factors, and chaperons, including ABC transporters, HSP20, and chaperonin GroEL, had a highly positive correlation with Claroideoglomus (Figure 4). Salt-tolerant rice rhizosphere-enriched bacteria, such as Pseudohongiella, Prolixibacter, Aquisphaera, and Dysgonomonas, were positively correlated with most DEGs of the three gene modules aforementioned (Figure 4). Furthermore, sugar ABC (*H2__13579_1*, *H2__18718_1*) and chaperonin GroEL (*H3__15385_1*) had a highly positive correlation with Aquisphaera. ABC transporter (*H2__16650_1*), efflux RND transporter (*H2__13298_1*, *H2__15663_1*), and transcriptional factors (*H2__4199_1*) were also positively correlated with Prolixibacter (Figure 4).

**Figure 5:**
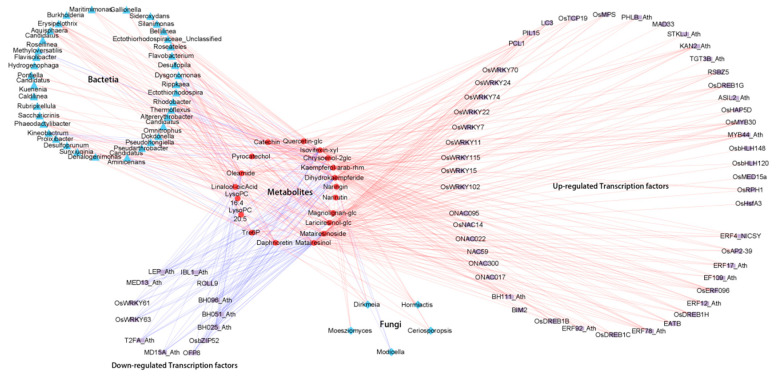
Multi-omics network of the comprehensive associations among genes, metabolites, and microorganisms. Rectangles represent genes, circles represent rice metabolites, triangles represent bacteria, and diamonds represent fungi. The gray and blue lines represent positive and negative correlations, respectively; the thickness of the line indicates the strength of the correlation. The thicker the line, the stronger the correlation. The 18 metabolites in rice were derived from the previous study [29], and the detailed correlation data of metabolites, genes, and microorganisms are presented in Table S7.

To further study the interaction between the plant and microbiome influencing salt tolerance, 18 differentially accumulated metabolites reported previously in salt-tolerant rice [29] were employed to calculate their correlation with rhizospheric microbiota. Using the stringent coefficient correlation (|PCC| > 0.80 and *p*-value < 0.05), 38 bacteria and 5 fungi were highly correlated with the 18 metabolites specifically accumulated in salt-tolerant rice (Table S7). Through further integrating the 63 rice transcription factors differentially expressed in salt-tolerant rice [29], this study presented the complex regulatory network for DAMs, DEGs, and microbial changes between rice varieties that differed in salt tolerance. From the perspective of rhizosphere microorganisms, fungi such as Ceriosporopsis and Dirkmeria were positively correlated with flavonoids, including chrysoeriol-2glc, isovitexin-xyl, dihydrokaempferide, and kaempferol-3-O-arabinoside-7-O-rhamnoside. Bacteria such as Desulfoprunum, Rippkaea, and Bellilinea were positively correlated with the flavonoids (dihydrokaempferide, isovitexin-8-O-xyloside, chrysoeriol-8-C-glucoside-7-O-(6″-feruloyl) glucoside) (Figure 5; Table S7).

## 4. Discussion

The intricate structural and functional dynamics of root-associated microbial communities play a pivotal role in orchestrating plant growth and performance [46,47]. The plant–microbiota interaction confers adaptive advantages to plant hosts by enhancing growth, nutrient uptake, stress tolerance, and pathogen resistance [48,49]. This complex interplay involves a network of genetic, biochemical, physical, and metabolic interactions between microbial communities and the environment, shaping the composition of plant-related microbiomes and modulating their beneficial traits such as nutrient acquisition and plant health [50]. The core mangrove microbiome unveils shared taxa potentially implicated in nutrient cycling and enhancing host survival [51]. The diversity and structure of rhizosphere microorganisms can be altered by the different types and quantities of metabolites produced by plants or microorganisms, potentially enhancing the host’s resistance to stress [52]. As soil salinization poses a significant challenge to rice production, enhancing rice salt tolerance has emerged as a critical objective in rice breeding efforts.

### 4.1. Microbial Community Structure and Function in Salt-Tolerant Rice Rhizosphere

Our phylogenetic analyses revealed a consortium of fungal and bacterial species thriving in the rhizosphere of salt-tolerant rice varieties. Through LEfSe analysis, we discerned that salt-tolerant rice rhizosphere harbored five predominant fungal species, namely *Hormiactis*, *Emericellopsis*, *Ceriosporopsis*, *Dirkmeia*, and *Moesziomyces*, belonging to the *Ascomycota* and *Basidiomycota* (Figure 2; Table 1). Notably, *Emericellopsis* species exhibited a penchant for saline environments, demonstrating robust adaptability and prevalence in such conditions [53]. Furthermore, it was shown that the *Emericellopsis cladophorae* strain MUM 19.33 exhibited a diverse array of enzymatic activities, including proteinases, cellulases, chitinases, pectinases, pectin lyases, and ureases. Importantly, all these enzymatic activities were found to be influenced by salt levels, underscoring the intricate interplay between microbial functionality and environmental salinity [54]. 

In the bacterial rhizosphere communities, our analysis spotlighted 12 genera, notably *Desulfoprunum*, *Sideroxydans*, *Hydrogenophaga*, *Candidatus Kuenenia*, *Pontiella*, *Rippkaea*, *Caldilinea*, *Bellilinea*, *Phaeodactylibacter*, *Flavisolibacter*, *Sunxiuqinia*, and *Prolixibacter*, as predominant genera in the rhizosphere of salt-tolerant rice in saline conditions (Figure 2b; Table 2). Plant microbiomes from saline environments could mitigate salt stress by direct mechanisms involved in protecting the plants (ACC deaminase, Exopolysaccharides (EPS), phytohormone production) or by indirect mechanisms inducing signaling and modifying the plant metabolome [14,15,55]. The recruitment of endophytic species such as Hydrogenophaga, influenced by melatonin and dopamine, underscores their role in enhancing plant physiological resilience under submersion stress [56]. Flavisolibacter spp. isolates, acting as plant growth-promoting rhizobacteria (PGPB) (by producing indole acetic acid and solubilizing phosphate), are capable of Cd-biosorption, which reduces Cd-uptake by tomato plants. Furthermore, the versatile Flavisolibacter spp. isolates exhibit multifaceted capabilities through the production of indole acetic acid and phosphate solubilization [57]. In saline environments, the resilience of Phaeodactylibacter underscores their adaptability to high-salt conditions, suggesting that the salinity levels in the wastewater studied foster the enrichment of salt-tolerant heterotrophic bacteria in the membrane bioreactor (MBR) settings [58,59]. The remarkable adaptability of Candidatus Kuenenia to high salinity levels, coupled with its unique ATP generation mechanism via a sodium-motive force mediated by Na^+^-pumping ATP hydrolase, underscores its resilience in challenging environments [60]. *Pontiella agarivorans* sp. represents a group of bacteria that may play an important role in the degradation of macroalgal polysaccharides, with relevance to the biogeochemical cycling of carbon, sulfur, and nitrogen in marine environments [61]. 

The findings of this study have potential applications in agricultural practices. By manipulating the rhizospheric microbial communities, it may be possible to enhance salt tolerance in crops, improving crop yields and food security in saline environments. Further studies are needed to validate the specific roles of identified microbial taxa in enhancing salt tolerance.

### 4.2. Correlation Analysis of DEGs and Rhizospheric Microbial Taxa

The rhizospheric microbiome acts as a salinity-alleviating agent; genes related to nutrients and salinity stress alleviation in PGPB were also identified [13,14]. Among the 7192 DEGs in rhizospheric microbiota, 274 transporters and 113 transport regulators were upregulated (Tables S4 and S5). Moreover, KEGG and GO enrichment analyses unveiled that a significant proportion of DEGs were enriched in pathways associated with the “two-component system”, “sulfur metabolism”, and “response to alkyl hydroperoxide” (Figure 3b,c), indicating that microorganisms may instigate genetic alterations and collectively contribute to salt tolerance, emphasizing the intricate interplay between plants and their microbial environment from a metatranscriptomic perspective. Microbial sulfur metabolism is tightly interwoven with biogeochemical cycles of important elements such as carbon, nitrogen, and iron, and has profound environmental implications [62,63]. In this study, all Sox proteins (sulfur oxidation c-type cytochrome SoxA and SoxX, sulfur-oxidizing protein, and thiosulfate oxidation carrier protein SoxY and SoxZ) were uniformly upregulated in the rhizospheric microbiome of salt-tolerant rice; this was also the case for DEGs in the “two-component system” category, including all chemotaxis proteins, the phosphate ABC transporter substrate-binding proteins PstS and PhoT, sugar ABC transporters, and two-component system response regulators OmpR and QseB (Table 3). Microbial two-component systems regulate a large array of fundamental processes. These include adaptations to changes in the environment (osmolality, light, temperature, oxygen), nutrient acquisition, and metabolism [64]. These indicate that microbes reduce stress in plants by controlling nutritional and hormonal equilibria and inducing systemic tolerance to stress [13,14].

To unravel the intricate interplay between rhizosphere microorganisms and salt stress, we embarked on a detailed analysis by constructing a correlation network that connected DEGs with specific microbial taxa (Figure 4; Table S6). In the face of diverse environmental challenges, microorganisms sense their surroundings accurately with intricate responses orchestrated by transcriptional factors that activate stress-related genes [65]. One noteworthy finding from our research was the prevalence of upregulated DEGs associated with salt tolerance, particularly highlighting the significance of ABC transporters. The ribose transporter complex (RbsABC), a member of the ABC importer family, facilitates the translocation of ribose across the inner membrane of *E. coli* by harnessing ATP as the primary energy source [66]. Bacteria employ distinct mechanisms for phosphate transport, with the high-affinity phosphate transport system PstSCAB and low-affinity PitH transporters playing crucial roles in regulating inorganic phosphate uptake. The utilization of the high-affinity system is particularly pronounced in habitats or environmental conditions where phosphate availability is limited [67,68]. Furthermore, we observed a heightened expression of chaperonin GroEL in salt-tolerant rice rhizosphere-enriched fungi and bacteria, underscoring their crucial role in adapting to saline conditions (Figure 4). GroEL has emerged as a prominent chaperonin protein, widely utilized for both in vitro refolding of aggregated proteins and in vivo solubilizing of aggregation-prone proteins, showcasing its utility in therapeutic and biotechnological applications [69]. The metatranscriptome and microbiome analyses indicated that the recruitment of rhizosphere microorganisms displayed striking variations in abundance and diversity between rice varieties of different salt tolerance. However, the specific mechanisms of how transporters in microbiota are regulated by salt stress warrants further investigation. 

### 4.3. Alterations of Rice Flavonoid Accumulation Mediated by Rhizosphere Microorganisms

Flavonoids are involved in a variety of biological activities in plants, which can protect plants from different biotics, including plant-parasitic nematodes, fungi and bacteria, and abiotic stresses, such as salt stress, drought stress, and higher and lower temperatures [69]. Chrysoeriol-7, a flavonoid isolated from rice, has been shown to act as a pesticide that can replace the use of chemical pesticides in rice farming because of its antifungal activities against *Fusarium gramibearum* and *Pythium graminicola* [70]. Isovitexin-8-O-xyloside is a plant stress substance with antioxidant and anti-inflammatory effects, which can help plants increase their resistance and adaptability in the face of environmental stress [71]. The concentrations of catechin, quercetin, luteolin 7-O-glucoside, and apigenin 7-O-glucoside in olive leaves increased after drought treatments, indicating that these phenolic compounds reduced the oxidative damage caused by water deficit stress [72].

In our previous metabolomic research, flavonoids, including dihydrokaempferide, isovitexin-8-O-xyloside, and catechin and lignans, such as matairesinol and matairesinoside, as well as trehalose-6-phosphate, were upregulated in salt-tolerant rice [29]. Flavonoids (rutin, naringenin, quercetin, and catechin) play a significant role in ROS scavenging for better redox regulation under salinity stress; therefore, the accumulation of flavonoid compounds and choline occurs in higher concentrations in the salt-tolerant genotype of rice under salinity stress [73]. Moreover, for the multi-omics network involving comprehensive associations among genes, metabolites, and microorganisms, the analysis has identified key transcription factor families, including 6 members of the NAC family and 13 members of the AP2/ERF family. Upregulated WRKY transcription factors were found to be positively correlated with specific metabolites, including three flavonoid metabolites and lignans/coumarins [74] (Figure 5). Salt stress triggers the accumulation of certain compounds in *Dendrobium officinale* leaves, particularly flavonoids, sugars, and alkaloids, which contribute to the salt-stress responses in leaf tissues of *D. officinale* [75]. In this study, from the microbe–phytometabolome association perspective, fungi such as *Ceriosporopsis* and *Dirkmeria* were positively correlated with the synthesis of flavonoids, including chrysoeriol-2glc, isovitexin-xyl, dihydrokaempferide, and kaempferol-arab-rhm (Figure 5). Bacteria such as *Desulfoprunum, Rippkaea*, and *Bellilinea* were positively correlated with the synthesis of flavonoids (Figure 5). The multi-omics network underscores the common metabolome target of rice transcription factors and the rhizospheric microbiome in plant response to salt stress, particularly in how they enhance plant adaptability by regulating the biosynthesis of specific metabolites (Figure 5). However, the mechanisms by which rhizosphere microorganisms and rice transcription factors regulate plant metabolite synthesis in parallel or consecutively remain to be elucidated. Investigating the signaling pathways and molecular interactions involved will provide a deeper understanding of the microbial–plant interface under salinity stress.

## 5. Conclusions

Through microbial amplicon sequencing and metatranscriptome analysis, we characterized the rhizosphere microbiome composition in the salt-tolerant rice TLJIAN, revealing distinct microbial profiles compared to the salt-sensitive HJING. The metatranscriptomic analysis identified 7192 DEGs in the two rice varieties, and the majority of DEGs were associated with a “two-component system”, “sulfur metabolism”, and “microbial metabolism in diverse environments”. Furthermore, correlation analysis of DEGs and microbial taxa revealed the upregulation of transporters, transcriptional factors, and chaperones in the rhizosphere fungi and bacteria of salt-tolerant rice, suggesting their potential role in salt-tolerance mechanisms. The multi-omics network of associations among rice genes, metabolites, and microorganisms revealed the comprehensive interaction between rice and microorganisms in response to salt stress. Fungi such as *Ceriosporopsis* and *Dirkmeria*, and bacteria such as *Desulfoprunum, Rippkaea,* and *Bellilinea* were positively correlated with the synthesis of flavonoids in salt-tolerant rice. Under salt stress conditions, plant and recruited microbial communities might coordinately regulate the expressions of transcription factors and transport proteins to modulate the synthesis and accumulation of metabolites. Further identifying the relevant microorganisms and elucidating their recruitment pathways is paramount to understanding the mechanisms that underlie salt tolerance in rice.

## Figures and Tables

**Table 1:** The fungal species enriched in rice rhizosphere.

Species	Group	LDA	*p*-Value
*Ascomycota.c__norank.o__norank.f__norank.g__Hormiactis.s__Hormiactis_candida*	TLJIAN	3.58	0.05
*Ascomycota.c__Sordariomycetes.o__Hypocreales.f__norank.g__Emericellopsis.s__Emericellopsis_microspora*		3.72	0.05
*Ascomycota.c__Sordariomycetes.o__Microascales.f__Halosphaeriaceae.g__Ceriosporopsis.s__Ceriosporopsis_halima*		3.50	0.04
*Basidiomycota.c__Ustilaginomycetes.o__Ustilaginales.f__Ustilaginaceae.g__Dirkmeia.s__Dirkmeia_churashimaensis*		3.45	0.05
*Basidiomycota.c__Ustilaginomycetes.o__Ustilaginales.f__Ustilaginaceae.g__Moesziomyces.s__Moesziomyces_antarcticus*		4.23	0.05
*Ascomycota.c__Sordariomycetes.o__Ophiostomatales.f__Ophiostomataceae.g__Raffaelea.s__Raffaelea_arxii*	HJING	3.96	0.04
*Basidiomycota.c__Agaricomycetes.o__Cantharellales.f__Hydnaceae.g__Hydnum.s__Hydnum_sp__2_RG_2012*		3.92	0.04
*Chytridiomycota.c__Chytridiomycetes.o__Cladochytriales.f__Nowakowskiellaceae.g__Nowakowskiella.s__Nowakowskiella_multispora*		3.71	0.05
*Mucoromycota.c__Glomeromycetes.o__Glomerales.f__Claroideoglomeraceae.g__Claroideoglomus.s__Claroideoglomus_sp_*		3.93	0.03
*Mucoromycota.c__Mortierellomycetes.o__Mortierellales.f__norank.g__Modicella.s__Modicella_reniformis*		3.64	0.05
*Zoopagomycota.c__Basidiobolomycetes.o__Basidiobolales.f__Basidiobolaceae.g__Schizangiella.s__Schizangiella_sp__ARSEF_2237*		4.96	0.05

**Table 2:** The bacteria enriched in rice rhizosphere.

Species	Group	LDA	*p*-Value
*Proteobacteria.c__Gammaproteobacteria.o__Xanthomonadales.f__Rhodanobacteraceae.g__Dokdonella.s__Dokdonella_koreensis*	TLJIAN	2.61	0.04
*Proteobacteria.c__Gammaproteobacteria.o__norank.f__norank.g__Pseudohongiella.s__Pseudohongiella_spirulinae*		2.56	0.05
*Proteobacteria.c__Deltaproteobacteria.o__Desulfobacterales.f__Desulfobulbaceae.g__Desulfoprunum.s__uncultured_bacterium*		2.96	0.05
*Proteobacteria.c__Betaproteobacteria.o__Nitrosomonadales.f__Gallionellaceae.g__Sideroxydans*		3.30	0.05
*Proteobacteria.c__Betaproteobacteria.o__Nitrosomonadales.f__Gallionellaceae.g__Gallionella*		2.98	0.05
*Proteobacteria.c__Betaproteobacteria.o__Burkholderiales.f__norank.g__Roseateles.s__Roseateles_terrae*		2.93	0.05
*Proteobacteria.c__Betaproteobacteria.o__Burkholderiales.f__Comamonadaceae.g__Hydrogenophaga.s__Hydrogenophaga_borbori*		3.40	0.05
*Proteobacteria.c__Betaproteobacteria.o__Burkholderiales.f__Burkholderiaceae.g__Burkholderia*		2.58	0.05
*Proteobacteria.c__Alphaproteobacteria.o__Sphingomonadales.f__Erythrobacteraceae.g__Altererythrobacter*		2.54	0.04
*Proteobacteria.c__Alphaproteobacteria.o__Hyphomicrobiales.f__Bradyrhizobiaceae.g__Bradyrhizobium.s__Bradyrhizobium_canariense_A*		2.82	0.05
*Planctomycetes.c__Planctomycetia.o__Pirellulales.f__Pirellulaceae.g__Rubripirellula.s__Rubripirellula_sp002689655*		2.60	0.05
*Planctomycetes.c__Planctomycetia.o__Pirellulales.f__Pirellulaceae.g__Pirellula.s__Pirellula_staleyi*		2.88	0.04
*Planctomycetes.c__Planctomycetia.o__Pirellulales.f__Pirellulaceae.g__Bremerella.s__Bremerella_volcania*		2.55	0.03
*Planctomycetes.c__Candidatus_Brocadiae.o__Candidatus_Brocadiales.f__Candidatus_Brocadiaceae.g__Candidatus_Kuenenia.s__Kuenenia_stuttgartiensis*		3.03	0.05
*Kiritimatiellaeota.c__Kiritimatiellae.o__Kiritimatiellales.f__Pontiellaceae.g__Pontiella.s__Pontiella_desulfatans*		3.13	0.05
*Firmicutes.c__Erysipelotrichia.o__Erysipelotrichales.f__Erysipelotrichaceae.g__Erysipelothrix.s__Erysipelothrix_sp011301115*		2.54	0.03
*Cyanobacteria.c__norank.o__Chroococcales.f__Aphanothecaceae.g__Rippkaea*		3.82	0.05
*Chloroflexi.c__Candidatus_Thermofonsia.o__norank.f__norank.g__Candidatus_Roseilinea.s__Roseilinea_sp003696945*		2.75	0.05
*Chloroflexi.c__Caldilineae.o__Caldilineales.f__Caldilineaceae.g__Caldilinea.s__Caldilinea_aerophila*		3.07	0.05
*Chloroflexi.c__Anaerolineae.o__Anaerolineales.f__Anaerolineaceae.g__Bellilinea*		3.50	0.05
*Candidatus_Omnitrophica.c__norank.o__norank.f__norank.g__Candidatus_Omnitrophus.s__uncultured_bacterium*		2.84	0.05
*Bacteroidetes.c__Saprospiria.o__Saprospirales.f__Haliscomenobacteraceae.g__Phaeodactylibacter.s__uncultured_bacterium*		3.19	0.05
*Bacteroidetes.c__Flavobacteriia.o__Flavobacteriales.f__Flavobacteriaceae.g__Flavobacterium.s__Flavobacterium_sp001898245*		2.65	0.03
*Bacteroidetes.c__Chitinophagia.o__Chitinophagales.f__Chitinophagaceae.g__Flavisolibacter.s__uncultured_bacterium*		3.14	0.05
*Bacteroidetes.c__Bacteroidia.o__Marinilabiliales.f__Prolixibacteraceae.g__Sunxiuqinia.s__uncultured_bacterium*		3.09	0.05
*Bacteroidetes.c__Bacteroidia.o__Marinilabiliales.f__Prolixibacteraceae.g__Prolixibacter.s__Prolixibacter_sp009617875*		3.16	0.05
*Proteobacteria.c__Deltaproteobacteria.o__Desulfobacterales.f__Desulfobulbaceae.g__Desulfopila.s__Desulfopila_sp005116675*	HJING	2.67	0.04
*Proteobacteria.c__Deltaproteobacteria.o__Desulfobacterales.f__Desulfobulbaceae.g__Desulfopila.s__Desulfopila_sp005116655*		2.71	0.03
*Planctomycetes.c__Planctomycetia.o__Pirellulales.f__Pirellulaceae.g__Blastopirellula.s__uncultured_bacterium*		2.83	0.04
*Firmicutes.c__Clostridia.o__Eubacteriales.f__Clostridiaceae.g__Clostridium.s__Clostridium_neonatale*		2.83	0.03
*Candidatus_Aminicenantes.c__norank.o__norank.f__norank.g__Candidatus_Aminicenans.s__Aminicenans_sakinawicola*		2.57	0.05
*Bacteroidetes.c__Bacteroidia.o__Bacteroidales.f__Dysgonomonadaceae.g__Dysgonomonas*		2.70	0.03

**Table 3:** DEGs associated with nutrient acquisition, signaling, and stress tolerance.

GeneID	log_2_FC	*p*-Value	Function
*ko00920 Sulfur metabolism*			
*H3__3700_2* *	1.5	0.00	Adenylyl-sulfate reductase subunit beta [*Candidatus Thiodiazotropha* endoloripes]
*H3__1908_1* *	5.1	0.00	Dissimilatory sulfite reductase beta subunit [*Candidatus Electrothrix* marina]
*H2__512_2*	3.6	0.00	Polysulfide reductase NrfD [*Anaeromyxobacter* sp. Fw109-5]
*H1__14555_1*	3.4	0.00	Pyridine nucleotide–disulfide oxidoreductase [*Gallionellales* bacterium GWA2_59_43]
*H2__2750_1*	12.2	0.00	Pyridine nucleotide–disulfide oxidoreductase [*gamma proteobacterium* symbiont of Ctena orbiculata]
*H2__7381_1* *	2.4	0.00	Pyridine nucleotide–disulfide oxidoreductase [*Hydrogenophaga* sp.]
*H1__15310_2*	2.1	0.00	Pyridine nucleotide–disulfide oxidoreductase [*Methylomonas* sp.]
*H1__7056_1*	1.2	0.01	Sulfide:quinone reductase [*Campylobacteraceae* bacterium 4484_4]
*H2__497_1*	3.2	0.00	Sulfide:quinone reductase [*Planctomycetes* bacterium]
*H1__9838_2*	3.5	0.00	Sulfide:quinone reductase [*uncultured* bacterium 9F08]
*H3__1113_1*	2.0	0.00	
*H3__7797_1*	3.3	0.00	
*H5__5333_1* *	−1.0	0.01	Sulfite reductase, dissimilatory-type subunit alpha [*Candidatus Thiodiazotropha* endoloripes]
*H2__4540_1*	2.1	0.00	Sulfur oxidation c-type cytochrome SoxA [*Gammaproteobacteria* bacterium HGW-Gammaproteobacteria-1]
*H2__6882_1*	1.3	0.00	
*H1__2376_1*	1.0	0.02	Sulfur oxidation c-type cytochrome SoxX [*Hydrogenophilales* bacterium 17-64-65]
*H1__9678_1*	1.2	0.02	Sulfur oxidation c-type cytochrome SoxX [*Hydrogenophilales* bacterium RIFOXYD1_FULL_62_11]
*H2__20025_1*	2.8	0.00	Sulfur oxidation protein [*Thiobacillus denitrificans* ATCC 25259]
*H1__13420_2*	2.5	0.00	Sulfur-oxidizing protein SoxY [*Thiohalomonas denitrificans*]
*H2__4540_3*	2.7	0.00	
*H3__1250_2*	3.6	0.00	Thiosulfate oxidation carrier protein SoxY [*Rhodocyclaceae* bacterium]
*H1__8087_2*	2.0	0.00	Thiosulfate oxidation carrier protein SoxY [*Sulfurivermis fontis*]
*H1__9678_2*	1.0	0.01	Thiosulfate oxidation carrier protein SoxY [*Thiobacillus* sp. 65-1402]
*H2__10327_2*	12.9	0.00	Thiosulfate oxidation carrier complex protein SoxZ [*Rhodocyclaceae* bacterium UTPRO2]
*H3__12915_2*	3.4	0.00	Thiosulfate oxidation carrier complex protein SoxZ [*Rhodocyclaceae* bacterium]
*H1__9678_3*	1.6	0.00	Thiosulfate oxidation carrier complex protein SoxZ [*Thiobacillus* sp. 63-78]
*H1__1575_2*	4.0	0.00	Sulfur compound chelating protein SoxZ [*Thioalbus denitrificans*]
*H2__18696_1*	2.3	0.00	Thiosulfohydrolase SoxB [*Gammaproteobacteria* bacterium HGW-Gammaproteobacteria-1]
*ko02020 Two-component system*			
*H3__19015_1*	1.7	0.00	Chemotaxis protein chev [*chromatiaceae* bacterium 2141t.stbd.0c.01a]
*H2__16504_2*	1.5	0.00	Chemotaxis protein chev [*sulfuriflexus mobilis*]
*H1__3069_1*	12.7	0.00	Chemotaxis protein chew [*ideonella dechloratans*]
*H2__15895_1*	1.6	0.00	Chemotaxis protein chew [*sulfurospirillum* sp. Uba12182]
*H3__17385_1*	3.1	0.00	Chemotaxis protein, partial [*gamma proteobacterium* symbiont of stewartia floridana]
*H1__6993_1*	1.7	0.00	Methyl-accepting chemotaxis protein [*cohaesibacter* sp. Cau 1516]
*H1__3694_1*	1.5	0.00	Methyl-accepting chemotaxis protein [*microvirgula aerodenitrificans*]
*H1__14709_1*	1.5	0.00	Methyl-accepting chemotaxis protein [*pseudomonas stutzeri*]
*H1__4784_1* *	4.1	0.00	Methyl-accepting chemotaxis protein PctA [*hydrogenophaga pseudoflava*]
*H1__503_1* *	2.0	0.00	DctP family TRAP transporter solute-binding subunit [*Hydrogenophaga* sp. PAMC20947]
*H2__20013_1*	1.3	0.00	Phosphate ABC transporter substrate-binding protein PstS [*Burkholderiales* bacterium RIFCSPLOWO2_12_FULL_67_210]
*H3__13953_1*	4.2	0.00	Phosphate ABC transporter substrate-binding protein PstS [*Gallionellaceae* bacterium]
*H3__4937_1*	1.9	0.00	Phosphate ABC transporter substrate-binding protein PstS [*Rugosibacter* sp.]
*H3__1560_1*	3.7	0.00	Phosphate ABC transporter substrate-binding protein PstS family protein [*Zooshikella ganghwensis*]
*H3__6928_1*	3.2	0.00	Phosphate ABC transporter substrate-binding protein, PhoT family [*Stigmatella aurantiaca*]
*H2__19436_1*	12.6	0.00	Sugar ABC transporter substrate-binding protein [*Cystobacter ferrugineus*]
*H2__5972_1*	5.0	0.00	Sugar ABC transporter substrate-binding protein [*Vitiosangium* sp. GDMCC 1.1324]
*H1__11617_1* *	1.9	0.00	Twitching motility protein [*Candidatus Propionivibrio* aalborgensis]
*H3__2461_1*	3.3	0.00	Two-component system response regulator OmpR [*macromonas* sp. Bk-30]
*H3__19062_1*	1.1	0.01	Two-component system response regulator OmpR [*propionivibrio limicola*]
*H3__15733_1*	1.8	0.00	Two-component system response regulator QseB [*sulfurirhabdus autotrophica*]
*ko01120 Microbial metabolism in diverse environments*			
*H3__1223_2* *	11.9	0.00	Methylglyoxal synthase [*Hydrogenophaga flava*]
*H1__12150_2* *	3.1	0.00	Nitrate reductase subunit alpha [*Hydrogenophaga flava*]
*H2__4699_1* *	4.8	0.00	
*H2__19965_1* *	2.1	0.00	
*H4__5995_1*	−0.9	0.01	Nitrite reductase [*bradyrhizobiaceae* bacterium]
*H3__12858_1*	3.8	0.00	Nitrogenase iron protein [*anaeromyxobacter* sp. Fw109-5]
*H2__15707_2*	3.7	0.00	Nitrogenase molybdenum-iron protein alpha chain [*deltaproteobacteria* bacterium]
*H3__12858_3*	4.1	0.00	
*Nitrogen metabolism* ^ @^			
*H3__3678_1*	8.0	0.00	Bifunctional nitrogenase iron-molybdenum cofactor biosynthesis protein NifEN [*deltaproteobacteria* bacterium]
*H2__18662_2*	5.9	0.00	
*H1__545_1*	4.4	0.00	Nitrogenase molybdenum-iron protein alpha chain [*geobacter metallireducens* rch3]
*H2__19381_1* *	2.4	0.00	Dinitrogenase iron-molybdenum cofactor biosynthesis protein [*candidatus bathyarchaeota* archaeon]
*H3__11121_2*	5.5	0.00	Nitrogen fixation protein FixH [*gammaproteobacteria* bacterium]
*H3__12858_1*	3.8	0.00	Nitrogenase iron protein [*anaeromyxobacter* sp. Fw109-5]
*H2__9290_1*	1.0	0.03	Nitrogen regulatory protein PII [*sulfuritalea hydrogenivorans* sk43h]
*Antioxidant defense* ^ @^			
*H4__1381_1*	−3.1	0.00	Catalase/peroxidase HPI [*dechloromonas* sp. Czr5]
*H3__1098_1*	0.9	0.03	Catalase/peroxidase HPI [*macromonas* sp. Bk-30]
*H1__7463_1*	0.8	0.03	Catalase/peroxidase HPI [*rhodocyclaceae* bacterium]
*H2__12915_2*	2.1	0.00	Glutathione peroxidase [*aliiglaciecola* sp. M165]
*H3__3081_1*	3.0	0.00	Glutathione peroxidase [*leptospira selangorensis*]
*H1__803_1*	1.6	0.00	Glutathione peroxidase [*pseudoalteromonas* sp. S554]
*H1__2271_1*	1.2	0.01	Peroxidase [*knoellia flava* tl1]
*H1__2240_1*	3.7	0.00	Thiol peroxidase [*chondromyces crocatus*]
*H3__13361_2*	1.9	0.00	Thioredoxin peroxidase [*candidate division* LCP-89 bacterium B3_LCP]
*H3__4643_1*	1.8	0.00	
*H1__614_1*	1.1	0.00	Fe-Mn family superoxide dismutase [*Sulfurisoma sediminicola*]
*H6__4570_1*	−2.1	0.00	Manganese superoxide dismutase [*Mariniradius saccharolyticus* AK6]
*H3__7603_1* *	1.5	0.00	Superoxide dismutase [Fe] [*Hydrogenophaga* sp. NH-16]
*H5__13305_1* *	1.9	0.00	Peroxiredoxin [*Candidatus Bathyarchaeota* archaeon]
*H1__13864_1* *	2.2	0.00	
*H3__6225_1* *	3.8	0.00	Peroxiredoxin BCP [*Hydrogenophaga pseudoflava*]
*H2__10642_2*	1.1	0.01	Peroxiredoxin family protein [*Gallionellaceae* bacterium]
*Osmoprotectants synthesis* ^ @^			
*H2__7719_1*	4.0	0.00	Arginine decarboxylase [*Myxococcales* bacterium]
*H2__3094_1*	2.7	0.00	Trehalose-phosphatase [*Myxococcaceae* bacterium]
*IAA production* ^ @^			
*H1__1517_1* *	12.5	0.00	Indole pyruvate ferredoxin oxidoreductase family protein [*Hydrogenophaga flava*]
*H3__18710_1* *	4.2	0.00	Indole pyruvate ferredoxin oxidoreductase family protein [*Hydrogenophaga pseudoflava*]
*H2__8783_1*	1.0	0.01	Indole pyruvate ferredoxin oxidoreductase family protein [*Macromonas* sp. BK-30]
*H3__11905_1*	3.2	0.00	Tryptophan synthase alpha chain [*Minicystis rosea*]

* DEGs in the predominant genera enriched in the rhizosphere of salt-tolerant rice TLJIAN. FC means the fold change of microbial genes in the rhizosphere of salt-tolerant rice against those in salt-sensitive rice. ^@^ The categories referred from the literature [14].

**Table 4:** The common DEGs of fungi and bacteria in the rhizosphere of salt-tolerant rice.

Gene ID	Annotation	log_2_FC	*p*-Value
Transporter			
*H5__1674_1*	ABC transporter substrate-binding protein	−1.18	0.00
*H6__11353_1*		−1.08	0.00
*H2__16650_1*		3.83	0.00
*H2__4757_1*	BMP family ABC transporter substrate-binding protein	4.03	0.00
*H3__11850_1*		1.42	0.00
*H6__7014_1*		−1.51	0.00
*H3__10157_1*	Multidrug ABC transporter permease/ATP-binding protein	−0.79	0.02
*H5__12143_1*	Peptide ABC transporter substrate-binding protein	−1.12	0.00
*H5__10216_1*		−1.52	0.00
*H5__4895_1*		−1.49	0.00
*H5__8560_1*		−1.31	0.00
*H3__12837_2*	Putrescine ABC transporter permease poth	1.50	0.00
*H2__13579_1*	Sugar ABC transporter substrate-binding protein	4.03	0.00
*H2__18718_1*		1.39	0.00
*H2__17137_1*	Anaerobic c4-dicarboxylate transporter, partial	−1.54	0.00
*H6__13585_1*	C4-dicarboxylate ABC transporter substrate-binding protein	−3.18	0.00
*H5__11644_1*	Glycerol-3-phosphate ABC transporter ATP-binding protein	−1.30	0.00
*H5__9522_1*	Carbohydrate ABC transporter substrate-binding protein	−1.09	0.00
*H5__148_1*		−1.64	0.00
*H2__15663_1*	Efflux RND transporter periplasmic adaptor subunit	5.40	0.00
*H2__8801_1*		1.93	0.00
*H2__13298_1*	Efflux RND transporter permease subunit	5.90	0.00
*H2__17837_1*	MFS transporter	2.71	0.00
*H5__9563_1*		−3.11	0.00
*H1__2962_1*	MMPL family transporter	2.16	0.00
Chaperon			
*H2__2356_1*	Heat shock hsp20	−1.34	0.00
*H6__11561_1*	Heat-shock protein hsp20	−3.17	0.00
*H6__14511_1*	Co-chaperone GroES	−1.95	0.00
*H1__13107_2*	Molecular chaperone DnaK	2.40	0.00
*H1__13994_1*	Hsp20/alpha-crystallin family protein	1.67	0.00
*H6__15143_1*		−1.99	0.00
*H2__21244_1*		2.90	0.00
*H2__21097_1*		−0.81	0.03
*H4__8752_1*		−3.18	0.00
*H1__11595_1*		2.45	0.00
*H1__4395_1*		1.93	0.00
*H1__13415_1*	Chaperonin GroEL	2.02	0.00
*H3__15385_1*		2.11	0.00
*H5__6447_1*		−2.59	0.00
*H2__9800_1*	Molecular chaperone GroEL	7.33	0.00
Transcriptional factor			
*H1__4420_3*	Copper-sensing transcriptional repressor csoR	2.74	0.00
*H2__684_1*	Fe-S cluster assembly transcriptional regulator IscR	−1.01	0.00
*H3__7060_2*	Helix-turn-helix transcriptional regulator	2.97	0.00
*H4__462_1*	Lrp/AsnC family transcriptional regulator, regulator for asnA, asnC, and gidA	−4.69	0.00
*H2__4199_1*	Transcriptional regulator	3.17	0.00
*H2__16343_1*	Transcriptional regulatory protein DegU	5.39	0.00
*H2__560_2*	Winged helix-turn-helix transcriptional regulator	3.74	0.00

## Data Availability

The sequence data used in this study were deposited in the BioProject database in NCBI (https://www.ncbi.nlm.nih.gov/bioproject (accessed on 28 September 2024)) under accession numbers PRJNA1101228 (16S rRNA), PRJNA1101242 (ITS), and PRJNA1103884 (metatranscriptome). All other data are contained within the main manuscript and Supplementary Materials.

## Supplementary Materials

The following supporting information can be downloaded at https://www.mdpi.com/article/10.3390/plants14010036/s1, Supplementary Table S1: Rhizospheric fungal difference between salt-tolerant rice TLJIAN and salt-sensitive HJING. Supplementary Table S2: Rhizospheric bacterial difference between salt-tolerant rice TLJIAN and salt-sensitive HJING. Supplementary Table S3: Microbial differentially expressed genes (DEGs) between salt-tolerant rice TLJIAN and salt-sensitive HJING rhizospheric metatranscriptome. Supplementary Table S4: Microbial transporters differentially expressed between salt-tolerant rice TLJIAN and salt-sensitive HJING rhizospheric metatranscriptome. Supplementary Table S5: Microbial transcription factors differentially expressed between salt-tolerant rice TLJIAN and salt-sensitive HJING rhizospheric metatranscriptome. Supplementary Table S6: Joint analysis of rhizosphere microbial community structure and metatranscriptome. Supplementary Table S7: The network of salt stress-responsive rice transcription factors, metabolites, and rhizospheric microbiota.
